# Preclinical atherosclerosis and metabolic syndrome increase cardio- and cerebrovascular events rate: a 20-year follow up

**DOI:** 10.1186/1475-2840-12-155

**Published:** 2013-10-23

**Authors:** Salvatore Novo, Angelica Peritore, Rosaria Linda Trovato, Francesco Paolo Guarneri, Daniela Di Lisi, Ida Muratori, Giuseppina Novo

**Affiliations:** 1 Chair of Cardiovascular Disease and Centre for the Early Diagnosis of Preclinical and Multifocal Atherosclerosis and for Secondary Prevention, Department of Internal Medicine and Specialties (DIBIMIS), University of Palermo, Via del Vespro n. 139-90127, Palermo, Italy; 2 Department of Internal Medicine and Cardiovascular Disease, Division of Cardiology, University Hospital “P. Giaccone”, Palermo, Italy

**Keywords:** Metabolic syndrome, Preclinical atherosclerosis, Intima-media thickness, Asymptomatic carotid plaque, Cardiac event, Cerebrovascular events

## Abstract

**Background:**

Intima-media thickness (IMT) is a validated marker of preclinical atherosclerosis and a predictor of cardiovascular events.

**Patients:**

We studied a population of 529 asymptomatic patients (age 62 ± 12.8 years), divided into two groups of subjects with and without Metabolic Syndrome (MetS).

**Methods:**

All patients, at baseline, have had a carotid ultrasound evaluation and classified in two subgroups: the first one without atherosclerotic lesions and the second one with preclinical atherosclerosis (increased IMT or asymptomatic carotid plaque). Cardiovascular endpoints were investigated in a 20-years follow-up.

**Results:**

There were 242 cardiovascular events: 144 among patients with MetS and 98 among in healthy controls (57.4% vs. 35.2%; P < 0.0001). 63 events occurred in patients with normal carotid arteries, while 179 events occurred in patients with preclinical atherosclerosis (31.8% vs. 54.1%; P < 0.0001). Of the 144 total events occurred in patients with MetS, 36 happened in the subgroup with normal carotid arteries and 108 in the subgroup with preclinical atherosclerosis (45% vs. 63.15%; P = 0.009). 98 events occurred in patients without MetS, of which 27 in the subgroup with normal carotid arteries and 71 in the subgroup with preclinical atherosclerosis (22.88% vs. 44.37%; P = 0.0003). In addition, considering the 63 total events occurred in patients without atherosclerotic lesions, 36 events were recorded in the subgroup with MetS and 27 events in the subgroup without MetS (45% vs. 22.88%; P = 0.0019). Finally, in 179 total events recorded in patients with preclinical carotid atherosclerosis, 108 happened in the subgroup with MetS and 71 happened in the subgroup without MetS (63.15% vs. 44.37%; P = 0.0009). The Kaplan-Meier function showed an improved survival in patients without atherosclerotic lesions compared with patients with carotid ultrasound alterations (P = 0.01, HR: 0.7366, CI: 0.5479 to 0.9904).

**Conclusions:**

Preclinical atherosclerosis leads to an increased risk of cardiovascular events, especially if it is associated with MetS.

## Introduction

The atherosclerotic process starts in childhood and proceeds silently over a long period of time before clinical manifestations. However, atherosclerosis is a significant cause of death in the developed countries and quite frequently it presents as a fatal event, hence the interest in detecting it in its subclinical stages [1].

Carotid artery Intima-Media Thickness (IMT) reflects the structural deterioration of the arterial wall, so it is considered a significant predictive marker of generalized atherosclerosis because of its correlation with coronary artery disease and it may predict future cardiovascular events in adults [1]. It is associated with established vascular risk factors (hypertension, dyslipidemia, diabetes mellitus, and smoking) or with less-conventional risk factors such as homocysteine, inflammation markers (C-reactive protein, fibrinogen) or uric acid [2-11].

The Metabolic Syndrome (MetS) represents a clustering of several cardiovascular (CV) risk factors including abdominal obesity, impaired glucose intolerance, dyslipidemia, and hypertension, with insulin resistance as a major characteristic [12-14]. MetS has got a high prevalence in the US population; in people >20 years old it is 24% and it rises to > 40% in patients ≥ 60 years of age [15,16]. Several clinical studies recognized MetS as responsible for the endothelial dysfunction, which is the first “step” in atherothrombotic disease and, in addition, it is observed the association between an increased Carotid intima-media thickness (C-IMT) and MetS [3,16-21]. The current study is the implementation of a study published in the month of August 2012 [22], with new data aiming to identify the influence of carotid preclinical atherosclerosis on prediction of cardiovascular events during a 20-years follow-up in a population of middle-aged subjects. We also evaluated the role of the metabolic syndrome on the risk of cardiovascular events comparing groups with preclinical atherosclerotic lesions.

## Methods

### Patients

**Figure 1:**
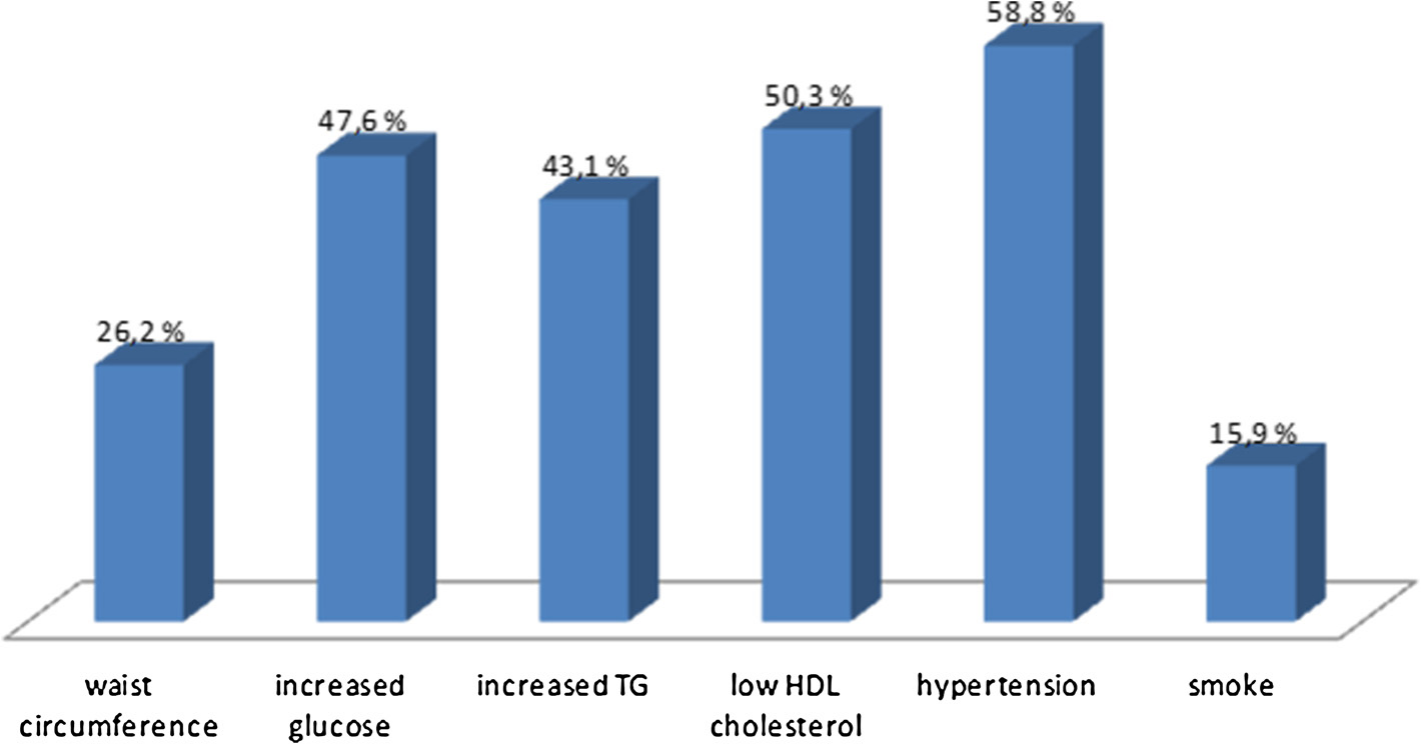
Prevalence of the cardiovascular risk factors in the study population.

Our study was performed on a population of 529 asymptomatic patients, aged between 25 and 87 years old (62 yrs ± 12.79) at baseline, divided in 257 male patients and 272 females, who were attending twenty years ago our “Centre for the Early Diagnosis of Preclinical and Multifocal Atherosclerosis and for the Cardiovascular Prevention”, in Palermo, Italy. We identified 529 asymptomatic subjects at baseline from our registry of more than 9000 patients referred from 1985 to 1991 and in follow-up in our centre. From this registry we selected the population evaluated in the present study with MetS, according to the document of Scientific International Societies - International Diabetes Federation (IDF), National Heart, Lung, and Blood Institute (NHLBI), World Hearth Federation, International Atherosclerosis Society e American Heart Association (AHA) – published in 2009 [23], which defined MetS as an alteration in 3 or more of the following 5 components: abdominal obesity, triglycerides, high-density lipoprotein (HDL) cholesterol, blood pressure (BP), and fasting plasma glucose. The following cut-off values were used to define alterations: waist circumference >102 cm for men and >88 cm for women for abdominal obesity, triglycerides ≥150 mg/dL (1.69 mmol/L), HDL-cholesterol <40 mg/dL (1.04 mmol/L) for men and < 50 mg/dL (1.29 mmol/L) for women, BP ≥ 130/≥85 mm Hg and fasting glucose ≥ 100 mg/dL. The choice of cutoff values for waist circumference was done according to the recent published consensus, taking into consideration the geographic distribution [23]. The most common CV risk factors (lifestyle, dietary habits, smoking, family and personal history of CV disease) were investigated. Moreover we evaluated the anthropometric parameters (weight, height, and waist circumference) and the laboratory ones (fasting triglycerides, HDL-c, fasting glucose and fasting insulin), as well as blood pressure. Figure 1 shows the prevalence of the mean cardiovascular risk factors in the population evaluated.

Body mass index (BMI) has been calculated as weight in kilograms divided by the square of height in meters. Waist circumference was calculated as the average of 2 measurements taken after inspiration and expiration at the midpoint between the lowest rib and iliac crest. Blood pressure (BP) measurements were performed with participants in the seated position and after a quiet resting period of 5 minutes. BP was measured in both arms with a random-zero mercury sphygmomanometer; the BP values used in this study are the average of the measurements on both the right and left arms. Pulse pressure was computed as the difference between the systolic.

BP (SBP) and the diastolic BP (DBP). Low-density lipoprotein (LDL) and high-density lipoprotein (HDL) fractions were separated from fresh serum by combined ultracentrifugation and precipitation; triglycerides were measured enzymatically. Blood glucose was measured using a glucose dehydrogenase method after precipitation of proteins by trichloroacetic acid.

We excluded patients affected by cancer, inflammatory bowel disease and with other autoimmune disorders because these diseases were considered as confounding variables for their ability to power the atherosclerosis process.

### Patient’s consent

Written informed consent was obtained from the patient for the publication of this report and any accompanying images.

Our research was carried out in compliance with the Helsinki Declaration and with international guidelines for the research on humans.

### Echo color Doppler examination of carotid arteries

Ultrasound examination was performed in all patients at baseline, in order to investigate the presence of preclinical atherosclerosis. B-mode real-time ultrasound was used to evaluate the arterial wall thickness in the carotid arteries using a machine Toshiba 270 SS with a probe of 7.5 to 10.0 MHz [24]. The power output, focus, depth of measurement, and gain were standardized by using the preset program incorporated within the software package of the ultrasound equipment. The IMT was defined as the distance between the echogenic line representing the intima- blood interface and the outer-echogenic line representing the adventitia junction. After freezing the image, the measurement was made with electronic calipers. Patients were examined in the supine position, and each carotid wall and segment was examined to identify the thickest intimal-medial site [25]. Three segments were identified and measured in anterior and posterior planes on each side: the distal 1 cm of the common carotid proximal to the bifurcation, the bifurcation itself, and the proximal 1 cm of the internal carotid artery. At each of these sites, we have determined the IMT, automatically measured, and detected any possible plaque. We primarily used the maximum carotid IMT value, which was determined as the mean of the maximum IMT of near- and far-wall measurements of both the left and right side arteries for each of the 3 arterial segments. If data on one of the walls or one of the sides were missing, maximum thickness of the available wall and side was used. The percentage of missing data was ≈ 35% (probably because of technical difficulties in the evaluation). Ultrasound examination was performed by one investigator, in blind and with no possibility of reproducing the IMT measurement. Carotid ultrasonography was performed by one sonographer to limit the risk of a large interobserver variability. However, for methodological correctness, the intraobserver agreement for sonographic measurement was calculated with a 4.1% to 5.0% coefficient of variation for repeated scans.

### Follow-up

Cardiovascular endpoints were investigated in a 20-years follow-up: acute myocardial infarction (AMI), angina pectoris, transient ischemic attack (TIA), ischemic stroke, abdominal aortic aneurysm (AAA), thromboendarterectomy (TEA) and cardiovascular death. Not fatal events were investigated through clinical controls during the follow-up in hospital. Fatal events were ascertained through the interrogation of family members or death certificates.

### Statistical analysis

Descriptive statistics were presented as percentages for categorical variables and as mean values ± standard deviation (SD) for continuous data. Differences between groups were compared by the *Chi-square test* for categorical variables. In addition, free-events survival was tested by *Kaplan-Meyer function* and *log-rank test.* A p-value < 0.05 was considered statistically significant. Statistical analysis was performed using the *Med Calc Program*.

## Results

Our population of 529 patients was divided into two categories, a first one with 251 patients suffering from metabolic syndrome and another one with 278 control subjects who had from 0 to 2 risk factors, so considered as controls. Both groups (participants with MetS and control subjects) were homogeneous about age, gender, smoking, diabetes and family history for CV diseases. The mean values of each component of MetS are showed in Table 1. According to the results of this ultrasound examination our patients were divided into three categories: 198 “normal subjects” (IMT < 0,9 mm), 162 patients with increased IMT (IMT > 0,9 mm and < 1,5 mm) and 169 patients with asymptomatic carotid plaque (IMT > 1,5 mm). In each of these groups, we identified two subgroups of subjects with or without MetS. Table 2 shows how it is more probable to find patients without MetS in the “normal subjects” subgroup (P = 0.015); on the contrary, in patients with increased IMT/asymptomatic plaque (331 patients) the presence of MetS is more frequent (P = 0.015).

**Table 1:** Mean values and standard deviation of the risk factors included in the metabolic syndrome cluster

	**MetS**	**Control subjects**	**P-value**
**BMI (Kg/m**^**2**^**)**	29.18±4.42	25.36±3.67	<0.0001
**Waist circumference (cm)**	90.80±7.99	85.09±9.41	<0.0001
**Fasting blood glucose (mg/dL)**	123.272±49.32	96.77±26.49	<0.0001
**Triglyceridemia (mg/dL)**	174.23±78.47	127.82±35.79	<0.0001
**HDL-cholesterol (mg/dL)**	40.24±13.004	47.79±11.14	<0.0001
**Systolic blood pressure (mmHg)**	150±1.2	125±0.09	<0.0001
**Diastolic blood pressure (mmHg)**	150±1.1	90±0.8	<0.0001

BMI: body mass index; HDL: high density lipoprotein cholesterol.

**Table 2:** Distribution of the study population according to the presence of metabolic syndrome and carotid ultrasound examinations

**IMT**	**MetS (n=251 patients)**	**Not MetS (n=278 patients)**	**P-value**	**Total (n=529 patients)**
**Normal**	80(31.87%)	118(42.5%)	P=0.015	198 patients
**Increased IMT/Asymptomatic plaque**	171(68.13%)	160(57.5%)	P=0.015	331 patients

MetS: metabolic syndrome; IMT: intima-media thickness. Normal IMT: < 0,9 mm; Increased IMT: > 0,9 mm and < 1,5 mm; Asymptomatic carotid plaque: IMT > 1,5 mm.

During the follow-up there were 242 cardiovascular events: 144 in patients with MetS (251 patients) and 98 in healthy controls (278 patients); (57.4% vs. 35.2%; P < 0.0001).

**Figure 2:**
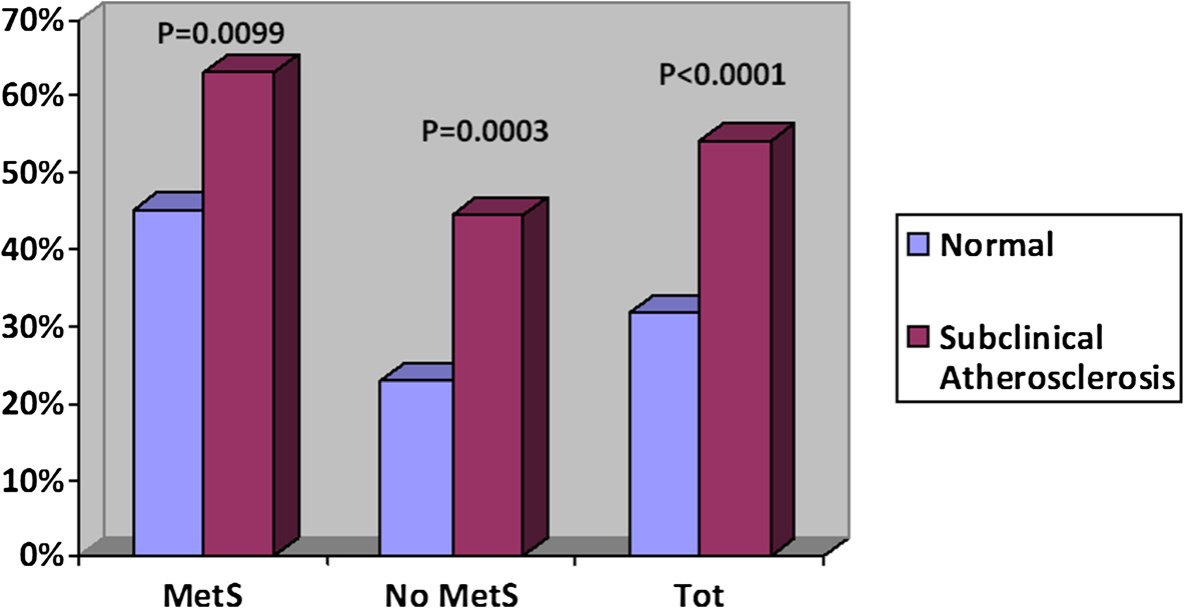
Comparison of incidence of events in the subgroups of study.

**Figure 3:**
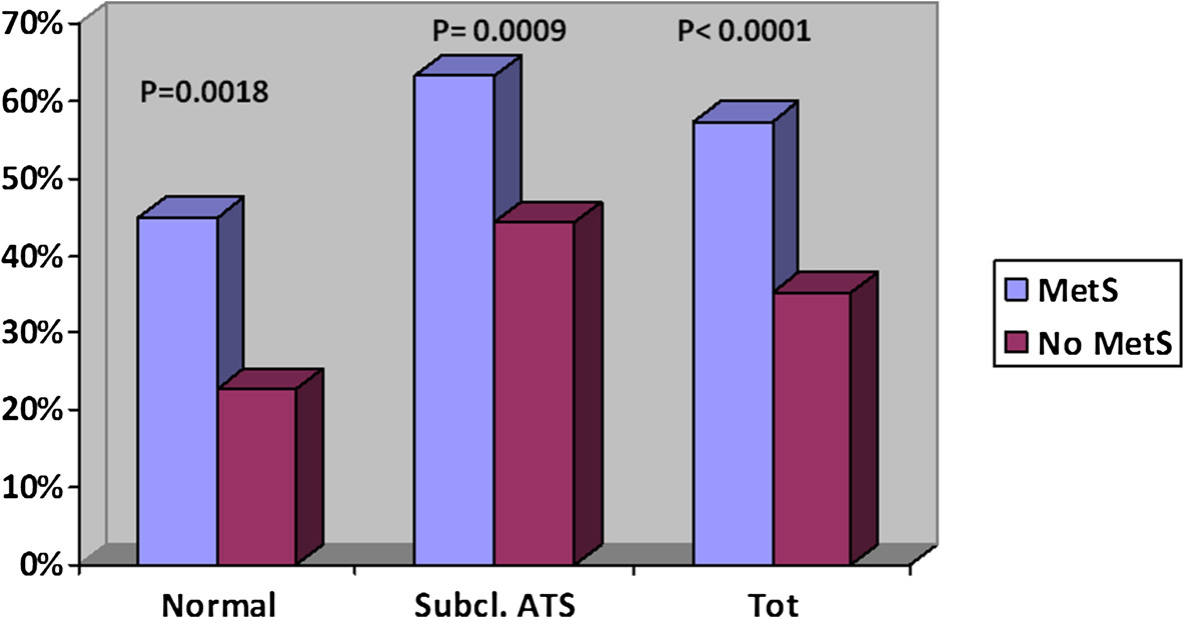
Distribution of cardiovascular events in relation to preclinical atherosclerosis and MetS.

As to the presence or absence of carotid preclinical atherosclerosis, 63 events occurred in patients with normal carotid arteries (198 patients), while 179 events occurred in patients with preclinical atherosclerosis (increased IMT/asymptomatic plaque; 331 patients); (31.8% vs. 54.1%; P < 0.0001). In the 144 total events occurred in patients with MetS, 36 happened in the subgroup of patients with normal carotid arteries and 108 happened in the subgroup of patients with preclinical atherosclerosis (45% vs. 63.15%; P = 0.0099). Similarly, in the 98 total events occurred in patients without MetS, 27 developed in the subgroup with normal carotid arteries and 71 in the subgroup with preclinical atherosclerosis (22.88% vs. 44.37%; P = 0.0003). So, preclinical atherosclerosis produced a significant increase of the risk of events, especially in presence of MetS (Table 3; Figure 2). In addition, in the 63 total events occurred in patients without atherosclerotic lesions, 36 events were recorded in the subgroup with MetS and 27 events in the subgroup without MetS (45% vs. 22.88%; P = 0.0018). Of the 179 total events recorded in patients with atherosclerotic lesions, 108 events happened in the subgroup with MetS and 71 events in the subgroup without MetS (63.15% vs. 44.37%; P = 0.0009). So, both in patients with preclinical atherosclerosis and in patients without atherosclerotic lesions the presence of MetS increased the risk of CV events (Table 3; Figure 3).

**Table 3:** Distribution of cardiovascular events in each subgroup of population

	**MetS (251 patients)**	**No MetS (278 patients)**	**P**
**Normal (198 patients)**	36 events/80 patients	27 events/118 patients	P=0.0018
	(45%)	(22.88%)	
**Subclinical atherosclerosis (331 patients)**	108 events/171 patients	71 events/160 patients	P=0.0009
	(63.15%)	(44.37%)	
**P**	P=0.0099	P=0.0003	

MetS: metabolic syndrome. Preclinical carotid atherosclerosis: increased IMT or asymptomatic carotid plaque.

Finally, comparing the subgroup without MetS and without atherosclerotic lesions to the subgroup affected by MetS and preclinical atherosclerosis, the second group showed a higher incidence of cardiovascular events (22.88% vs. 63.15%; P < 0.0001); (Table 3).

The cardiovascular events occurred in the groups can be summarized as follows (Table 4):

● In patients without preclinical atherosclerosis (normal; 198 patients) occurred: 12 cases of angina, 11 AMI, 13 TIAs, 14 strokes, 2 AAA, 1 TEA and 10 cardiovascular deaths.

● In patients with carotid ultrasound lesions (IMT or asymptomatic plaque; 331 patients) occurred: 21 cases of angina pectoris, 49 AMI, 35 TIAs, 33 strokes, 4 AAA, 4 TEA and 33 cardiovascular deaths.

**Table 4:** Events occurred in patients with and without carotid preclinical atherosclerosis

	**Angina**	**AMI**	**TIA**	**Stroke**	**AAA**	**TEA**	**Fatal events**	**Total events**
**Normal (198 patients)**	12(19%)	11(17.4%)	13(19%)	14(22.2%)	2(3.17%)	1(1.58%)	10(15.9%)	63(31.8%)
**IMT/Asymptomatic plaque (331 patients)**	21(20%)	49(27.3%)	35(20%)	33(24%)	4(3.23%)	4(2.23%)	33(18.4%)	179(54.1%)
**P**		**P=0.003**						**P<0.0001**

AMI: acute myocardial infarction; TIA: transient ischemic attack; AAA: abdominal aortic aneurysm; TEA: thrombo-endo-arterectomy.

**Figure 4:**
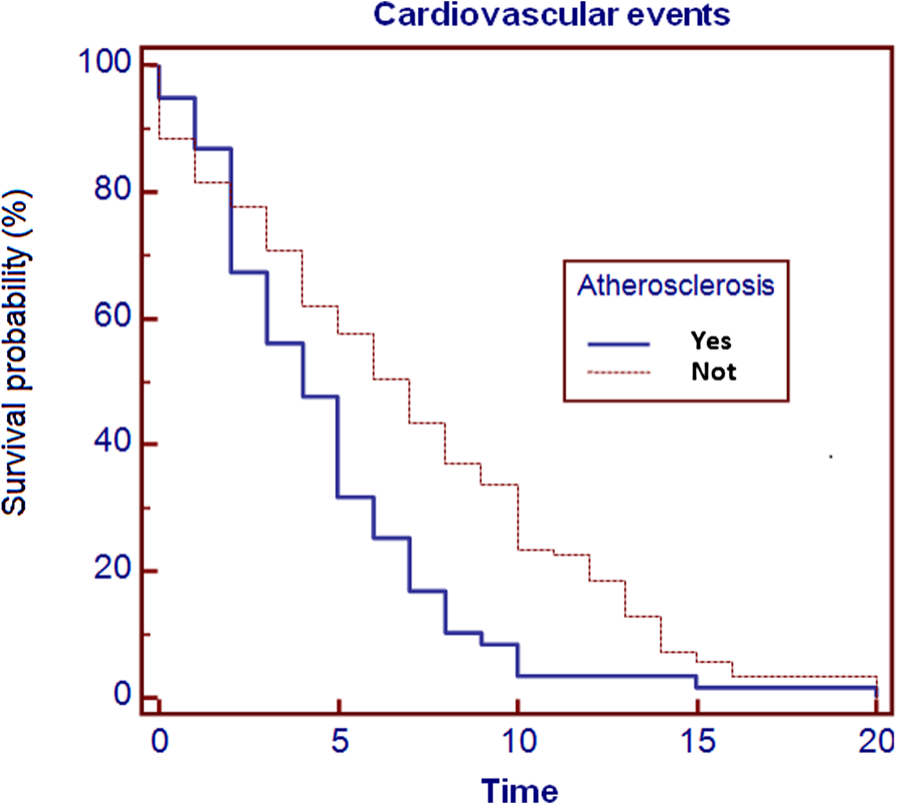
Event-free survival according to the Kaplan Meyer function.

In order to assess the role of preclinical atherosclerosis, the event-free survival was evaluated in patients with and without carotid ultrasound lesions by Kaplan Meyer function (Figure 4). It showed an improved survival in patients who had not atherosclerotic lesions (*P* = 0.0159, HR: 0.7366, CI: 0.5479-0.9904).

## Discussion

Cardiovascular diseases (CVD) are the leading cause of morbidity and mortality in Americans and Europeans. Signs of CVD are evident in the vessels before the onset of clinical disease symptoms. Subclinical disease can be measured using non-invasive B-mode ultrasound in order to assess common carotid artery intima-media thickness. Vascular aging is a natural process that can be modified by lifestyle and pharmacologic interventions. IMT is measured as the distance between the lumen-intimal interface and the medial-adventitial interface. The measurement varies depending on several factors: the used carotid artery (both the left and the right side or only one side); the artery segment(s) (common carotid, internal carotid, bulb); whether near and far wall images are used and whether plaque is included in the measurement. Typically IMT is the average of all available segments and sides. It is reliable, reproducible [26] and measured in a wide variety of populations [27-31]. Several studies have demonstrated the relationship between thicker IMT and/or carotid plaque and traditional cardiovascular risk factors like age, smoking, type 2 diabetes, hypertension, higher low density lipoprotein (LDL) cholesterol [27,31]. It has been also described that preclinical atherosclerosis is associated with type 1 diabetes in young adults [32] and with gestational diabetes [33,34], showing an increased C-IMT in these patients comparing it to controls.

IMT and carotid plaque are indicators of atherosclerosis and predictors of myocardial infarction and cardiovascular mortality [35,36]. A thicker IMT predicts coronary heart disease [28], stroke [29], and myocardial infarction [29,31]. A 5,5 years-follow-up showed that the risk of myocardial necrosis increases for about 10- 15% just if IMT increases for 0,1 mm while the risk of stroke increases for about 13- 18% [37]. Moreover other studies underlined that the Metabolic Syndrome could produce a more rapid deterioration of brain function, because it seems to be related to a low gray matter cerebral blood flow (GM-CBF) and to a lower immediate memory function if compared to control subjects [38].

The results of IMPROVE Study (Carotid Intima-Media Thickness [IMT] and IMT-Progression as Predictors of Vascular Events in a High Risk European Population) compared the performance of several measures of C-IMT as predictors of cardiovascular events and investigated whether they add to the predictive accuracy of Framingham risk factors. Therefore, this study suggests that a risk stratification strategy based on C-IMT and ICCAD (inter-adventitia common carotid artery diameter) as an adjunct to Framingham risk factors is a rational approach to prevent cardiovascular diseases [39].

Our data confirm the results of other clinical studies (nationals and internationals) that have examined the relationship between preclinical atherosclerosis, MetS and CV risk [40-43].

We demonstrated that the presence of preclinical atherosclerosis lead to an increased incidence of cardiovascular events, especially if it is associated with MetS. In fact, total cardiovascular events were more frequent in the group of patients with carotid preclinical atherosclerosis compared to patients with normal carotid arteries (P < 0.0001). Similarly, both in patients with MetS and in patients without MetS cardiovascular events were more frequent in presence of preclinical atherosclerosis than without these lesions. Moreover, also the importance of MetS resulted significant, in fact, both in patients with preclinical atherosclerosis and in patients without atherosclerotic lesions the presence of MetS increased the risk of CV events. Event-free survival was lower in patients with preclinical atherosclerosis than in healthy subjects (P = 0.0159, HR: 0.7366, CI 0.5479-0 0.9904).

Another study also described that progression of carotid preclinical atherosclerosis is greater especially in cases of MetS [44]. In a study conducted on 1442 men and 1532 women it was observed that subjects with MetS had higher levels of IMT and total plaque area (TPA) at follow up than those without MetS and that Mets predicted progression of IMT and TPA in patients below 50 years old [44].

According to the recent ESC 2012 guidelines on cardiovascular prevention, the detection of an asymptomatic carotid plaque put subjects in the very high risk category [43].

Therefore we suggest to investigate the presence of preclinical atherosclerosis in all patients > 45 years old by a carotid echo color Doppler test, because, in primary prevention, the IMT measurement can give further information for a better stratification of global cardiovascular risk [42,45]. We also recommend preventing the development of Metabolic Syndrome’s abnormalities, encouraging daily physical activity and Mediterranean diet and starting early pharmacological treatment of modifiable risk factors [46].

## Conclusions

Preclinical atherosclerosis leads to an increased risk of cardiovascular events, especially if it is associated with Metabolic Syndrome. Therefore, preclinical atherosclerosis added to traditional risk factors can improve the cardiovascular risk prediction [37,47], especially in presence of metabolic abnormalities.

## Abbreviations

IMT: Intima-media thickness; MetS: Metabolic syndrome; HDL: High-density lipoprotein; BP: Blood pressure; C-IMT: Carotid intima-media thickness; BMI: Body mass index; AMI: Acute myocardial infarction; TIA: Transient ischemic attack; AAA: Abdominal aortic aneurysm; TEA: Thromboendarterectomy; CVD: Cardiovascular diseases; LDL: Low density lipoprotein; ICCAD: inter-adventitia common carotid artery diameter.

## Competing interests

The authors declare that they have no competing interests.

## Authors’ contributions

SN: responsible for the critical review of the manuscript; AP: responsible for the follow-up of patients; RLT: responsible for writing the paper; FPG: responsible for statistical calculation and elaboration; DDL: responsible of the data base; IM, responsible for carotid ultrasound evaluation; GN: responsible of the ideation. All authors read and approved the final manuscript.

## References

[B1] Dahl-Jørgensen K Larsen JR Hanssen KF Atherosclerosis in childhood and adolescent type 1 diabetes: early disease, early treatment? Diabetologia 2005 12 1445 1453 15971059 10.1007/s00125-005-1832-1

[B2] Panayiotou AG Griffin M Kouis P Tyllis T Georgiou N Bond D Nicolaides AN Association between presence of the metabolic syndrome and its components with carotid intima-media thickness and carotid and femoral plaque area: a population study Diabetol Metab Syndr 2013 12 1 44 23962225 10.1186/1758-5996-5-44 PMC3765162

[B3] Koskinen J Kahonen M Viikari JSM Conventional CV risk factors and MetS in predicting carotid intima-media thickness progression in young adults. The CV risk in young Finns study Circulation 2009 12 229 236 19581494 10.1161/CIRCULATIONAHA.108.845065

[B4] Heiss G Sharrett AR Barnes R Carotid atherosclerosis measured by B-mode ultrasound in populations: associations with cardiovascular risk factors in the ARIC study Am J Epidemiol 1991 12 250 256 1877584 10.1093/oxfordjournals.aje.a116078

[B5] Lande MB Carson NL Roy J Effects of childhood primary hypertension on carotid intima media thickness. A matched controlled study Hypertension 2006 12 40 44 16735644 10.1161/01.HYP.0000227029.10536.e8

[B6] Puato M Palatini P Zanardo M Increase in carotid intima-media thickness in grade I hypertensive subjects. White-coat versus sustained hypertension Hypertension 2008 12 1300 1305 18378860 10.1161/HYPERTENSIONAHA.107.106773

[B7] Milio G Corrado E Novo S Asymptomatic carotid lesions and aging: Role of hypertension and other traditional and emerging risk factors Arch Med Res 2006 12 342 347 16513482 10.1016/j.arcmed.2005.06.012

[B8] Brohall G Oden A Fagerberg B Carotid artery intima-media thickness in patients with type 2 diabetes mellitus and impaired glucose tolerance: a systematic review Diabet Med 2006 12 609 616 16759301 10.1111/j.1464-5491.2005.01725.x

[B9] Linnebank M Moskau S Farmand S Homocysteine and carotid intima-media thickness in a German population: lack of clinical relevance Stroke 2006 12 2840 2842 17008631 10.1161/01.STR.0000244764.02851.d3

[B10] Thakore AH Guo CY Larson MG Association of multiple inflammatory markers with carotid intimal medial thickness and stenosis (from the Framingham Heart Study) Am J Cardiol 2007 12 1598 1602 17531588 10.1016/j.amjcard.2007.01.036

[B11] Tavil Y Kaya MG Oktar SO Uric acid level and its association with carotid intima-media thickness in patients with hypertension Atherosclerosis 2008 12 159 163 17416371 10.1016/j.atherosclerosis.2007.03.008

[B12] Third Report of the National Cholesterol Education Program (NCEP) Expert Panel on Detection, Evaluation, and Treatment of High Blood Cholesterol in Adults (Adult Treatment Panel III) final report Circulation 2002 12 3143 3421 12485966

[B13] Novo S Balbarini A Belch JJ The metabolic syndrome: definition, diagnosis and management Int Angiol 2008 12 220 231 18506125

[B14] Novo G Corrado E Muratori I Novo S Markers of inflammation and prevalence of vascular disease in patients with metabolic syndrome Int Angiol 2007 12 312 317 18091698

[B15] Ford E Giles W Dietz W Prevalence of the metabolic syndrome among U.S. adults: findings from the third National Health and Nutrition Examination Survey JAMA 2002 12 356 359 11790215 10.1001/jama.287.3.356

[B16] Reaven GM Banting lecture 1988: role of insulin resistance in human disease Diabetes 1988 12 1595 1607 3056758 10.2337/diab.37.12.1595

[B17] Kawamoto R Tomita H Inoue A Ohtsuka N Kamitani A Metabolic syndrome may be a risk factor for early carotid atherosclerosis in women but not in men J Atheroscler Thromb 2007 12 36 43 17332691 10.5551/jat.14.36

[B18] NcNeill AM Rosamond WD Girman CJ Prevalence of coronary heart disease and carotid thickening in patients with metabolic syndrome (the ARIC study) Am J Cardiol 2004 12 1249 1254 10.1016/j.amjcard.2004.07.107 15541239

[B19] Scuteri A Najjar SS Muller DC Andres Metabolic syndrome amplifies the age-associated increases in vascular thickness and stiffness J Am Coll Cardiol 2004 12 1388 1395 15093872 10.1016/j.jacc.2003.10.061

[B20] Tzou WS Douglas PS Srinivasan SR Increased preclinical atherosclerosis in young adults with metabolic syndrome: the Bogalusa heart study J Am Coll Cardiol 2005 12 457 463 16053958 10.1016/j.jacc.2005.04.046

[B21] Sipila K Moilanem L Nieminen T Metabolic syndrome and carotid intima media thickness in the Health 2000 Survey Atherosclerosis 2009 12 276 281 18848324 10.1016/j.atherosclerosis.2008.08.029

[B22] Novo S Peritore A Guarneri FP Metabolic syndrome (MetS) predicts cardio and cerebrovascular events in a twenty years follow-up. A prospective study Atherosclerosis 2012 12 468 472 22704563 10.1016/j.atherosclerosis.2012.05.018

[B23] Alberti KG Eckel RH Grundy SM Harmonizing the metabolic syndrome: a joint interim statement of the International Diabetes Federation Task Force on Epidemiology and Prevention; National Heart, Lung, and Blood Institute; American Heart Association; World Heart Federation; International Atherosclerosis Society; and International Association for the Study of Obesity Circulation 2009 12 1640 1645 19805654 10.1161/CIRCULATIONAHA.109.192644

[B24] Corrado E Rizzo M Novo S Markers of inflammation and infection influence the outcome of patients with baseline asymptomatic carotid lesions: a 5-year follow-up study Stroke 2006 12 482 486 16373649 10.1161/01.STR.0000198813.56398.14

[B25] Salonen R Haapanen A Salonen JT Measurement of intima media thickness of common carotid arteries with high resolution B-mode ultrasonography: inter- and intra-observer variability Ultrasound Med Biol 1991 12 225 230 1887507 10.1016/0301-5629(91)90043-v

[B26] Bots ML Grobbee DE Intima Media Thickness as a Surrogate Marker for Generalized Atherosclerosis Cardiovasc Drugs Therapy 2002 12 341 351 10.1023/a:1021738111273 12652104

[B27] Crouse JR Goldbourt U Evans G Risk Factors and Segment-Specific Carotid Arterial Enlargement in the Atherosclerosis Risk in Communities (ARIC) Cohort Stroke 1996 12 69 75 8553406 10.1161/01.str.27.1.69

[B28] Bots ML Hoes AW Koudstaal PJ Hofman A Grobbee DE Common carotid intima-media thickness and risk of stroke and myocardial infarction: the Rotterdam study Circulation 1997 12 1432 1437 9315528 10.1161/01.cir.96.5.1432

[B29] O’Leary DH Polak JF Kronmal RA Manolio TA Burke GL Wolfson SKJ Carotid-artery intima and media thickness as a risk factor for myocardial infarction and stroke in older adults. Cardiovascular health study collaborative research group Ann Internal Med 1999 12 14 22 10.1056/NEJM199901073400103 9878640

[B30] Urbina EM Srinivasan SR Tang R Bond MG Kieltyka L Berenson GS Study BH Impact of multiple coronary risk factors on the intima-media thickness of different segments of carotid artery in healthy young adults (the Bogalusa heart study) Am J Cardiol 2002 12 953 958 12398961 10.1016/s0002-9149(02)02660-7

[B31] Salonen JT Salonen R Ultrasound B-mode imaging in observational studies of atherosclerotic progression Circulation 1993 12 156 165 8443925

[B32] Rathsman B Rosfors S Sjöholm A Nyström T Early signs of atherosclerosis are associated with insulin resistance in non-obese adolescent and young adults with type 1 diabetes Cardiovasc Diabetol 2012 12 145 23185996 10.1186/1475-2840-11-145 PMC3538551

[B33] Sousa MA Guimarães IC Daltro C Guimarães AC Association between birth weight and cardiovascular risk factors in adolescents Arq Bras Cardiol 2013 12 9 17 23740400 10.5935/abc.20130114 PMC3998182

[B34] Freire CM Barbosa FB de Almeida MC Miranda PA Barbosa MM Nogueira AI Guimarães MM Nunes Mdo C Ribeiro-Oliveira A Jr Previous gestational diabetes is independently associated with increased carotid intima-media thickness, similarly to metabolic syndrome - a case control study Cardiovasc Diabetol 2012 12 59 22651701 10.1186/1475-2840-11-59 PMC3403942

[B35] Johnsen SH Mathiesen EB Joakimsen O Carotid atherosclerosis is a stronger predictor of myocardial infarction in study women than in Men: a 6-year follow-up study of 6226 persons: the tromso Stroke 2007 12 2873 2880 17901390 10.1161/STROKEAHA.107.487264

[B36] Rizzo M Corrado E Novo S Prediction of cardio- and cerebro-vascular events in patients with subclinical carotid atherosclerosis and low HDL-cholesterol Atherosclerosis 2008 12 389 395 18258237 10.1016/j.atherosclerosis.2007.12.020

[B37] Novo S Visconti CL Amoroso GR Asymptomatic carotid lesions add to CV risk prediction Eur J Cardiovasc Prev Rehabil 2010 12 514 518 20351551 10.1097/HJR.0b013e328337ccbd

[B38] Birdsill AC Carlsson CM Willette AA Okonkwo OC Johnson SC Xu G Oh JM Gallagher CL Koscik RL Jonaitis EM Hermann BP Larue A Rowley HA Asthana S Sager MA Bendlin BB Low cerebral blood flow is associated with lower memory function in metabolic syndrome Obesity (Silver Spring) 2013 12 1313 1320 23687103 10.1002/oby.20170 PMC3742665

[B39] Baldassare D Hamsten A Veglia F IMPROVE Study Group. Measurements of Carotid Intima- Media thickness and of Interadventitia Common Carotid Diameter Improve Prediction of Cardiovascular Events: Results of the IMPROVE (Carotid Intima Media Thickness and IMT progression as Predictors of vascular events in a high risk European Population) Study J Am Coll Cardiol 2012 12 1489 1499 10.1016/j.jacc.2012.06.034 22999719

[B40] Nambi V Chambless L Folsom AR Carotid intima-media thickness and presence or absence of plaque improves prediction of coronary heart disease risk: the ARIC (atherosclerosis Risk in Communities) study J Am Coll Cardiol 2010 12 55 1600 1607 20378078 10.1016/j.jacc.2009.11.075 PMC2862308

[B41] Sehestedt T Jeppesen J Hansen TW Risk prediction is improved by adding markers of subclinical organ damage to SCORE Eur Heart J 2010 12 883 891 20034972 10.1093/eurheartj/ehp546

[B42] Mookadam F Moustafa SE Lester SJ Warsame T Subclinical atherosclerosis: evolving role of carotid intima-media thickness Prev Cardiol 2010 12 186 197 20860643 10.1111/j.1751-7141.2010.00072.x

[B43] European Guidelines on cardiovascular disease prevention in clinical practice (version 2012): the Fifth Joint Task Force of the European Society of Cardiology and Other Societies on Cardiovascular Disease Prevention in Clinical Practice (constituted by representatives of nine societies and by invited experts) Eur J Prev Cardiol 2012 12 585 667 10.1177/2047487312450228 22763626

[B44] Herder M Arntzen KA Johnsen SH Mathiesen EB The metabolic syndrome and progression of carotid atherosclerosis over 13 years. The Tromsø study Cardiovasc Diabetol 2012 12 77 22738646 10.1186/1475-2840-11-77 PMC3539868

[B45] Novo S Carità P Corrado E Preclinical carotid atherosclerosis enhances the global cardiovascular risk and increases the rate of cerebro- and cardiovascular events in a five-year follow-up Atherosclerosis 2010 12 287 290 20171636 10.1016/j.atherosclerosis.2010.01.019

[B46] Laaksonen DE Lakka HM Salonen JT Niskanen LK Rauramaa R Lakka TA Low levels of leisure-time physical activity and cardiorespiratory fitness predict development of the metabolic syndrome Diabetes Care 2002 12 1612 1618 12196436 10.2337/diacare.25.9.1612

[B47] Shah PK Screening asymptomatic subjects for subclinical atherosclerosis: can we, does it matter, and should we? J Am Coll Cardiol 2010 12 98 105 20620724 10.1016/j.jacc.2009.09.081

